# Numerical Model for the Determination of Erythrocyte Mechanical Properties and Wall Shear Stress *in vivo* From Intravital Microscopy

**DOI:** 10.3389/fphys.2019.01562

**Published:** 2020-01-23

**Authors:** Vivek P. Jani, Alfredo Lucas, Vinay P. Jani, Carlos Munoz, Alexander T. Williams, Daniel Ortiz, Ozlem Yalcin, Pedro Cabrales

**Affiliations:** ^1^School of Medicine, Johns Hopkins University, Baltimore, MD, United States; ^2^Functional Cardiovascular Engineering, Department of Bioengineering, University of California, San Diego, San Diego, CA, United States; ^3^Department of Biomedical Engineering, Universidad de los Andes, Bogota, Colombia; ^4^Koc University School of Medicine, Istanbul, Turkey

**Keywords:** erythrocyte mechanics, wall shear stress, shear strain, capillary, plug flow, microcirculation, intravital microscopy

## Abstract

The mechanical properties and deformability of Red Blood Cells (RBCs) are important determinants of blood rheology and microvascular hemodynamics. The objective of this study is to quantify the mechanical properties and wall shear stress experienced by the RBC membrane during capillary plug flow *in vivo* utilizing high speed video recording from intravital microscopy, biomechanical modeling, and computational methods. Capillaries were imaged in the rat cremaster muscle pre- and post-RBC transfusion of stored RBCs for 2-weeks. RBC membrane contours were extracted utilizing image processing and parametrized. RBC parameterizations were used to determine updated deformation gradient and Lagrangian Green strain tensors for each point along the parametrization and for each frame during plug flow. The updated Lagrangian Green strain and Displacement Gradient tensors were numerically fit to the Navier-Lame equations along the parameterized boundary to determined Lame's constants. Mechanical properties and wall shear stress were determined before and transfusion, were grouped in three populations of erythrocytes: native cells (NC) or circulating cells before transfusion, and two distinct population of cells after transfusion with stored cells (SC1 and SC2). The distinction, between the heterogeneous populations of cells present after the transfusion, SC1 and SC2, was obtained through principle component analysis (PCA) of the mechanical properties along the membrane. Cells with the first two principle components within 3 standard deviations of the mean, were labeled as SC1, and those with the first two principle components greater than 3 standard deviations from the mean were labeled as SC2. The calculated shear modulus average was 1.1±0.2, 0.90±0.15, and 12 ± 8 MPa for NC, SC1, and SC2, respectively. The calculated young's modulus average was 3.3±0.6, 2.6±0.4, and 32±20 MPa for NC, SC1, and SC2, respectively. o our knowledge, the methods presented here are the first estimation of the erythrocyte mechanical properties and shear stress *in vivo* during capillary plug flow. In summary, the methods introduced in this study may provide a new avenue of investigation of erythrocyte mechanics in the context of hematologic conditions that adversely affect erythrocyte mechanical properties.

## 1. Introduction

Red Blood Cells (RBCs) are highly deformable biconcave particles with diameter 8 μm that constitute ~45% of total blood volume. The mechanical properties and deformability of RBCs are some of the most important determinants of blood rheology and microvascular hemodynamics, and decreased RBC deformability is characteristic of several hematologic disorders (Rand and Burton, 1964; Nash et al., 1984; Fedosov et al., 2014). RBC mechanical properties are derived from their membrane structure, which consists of a spectrin-actin cytoskeleton linked to the lipid bilayer and transmembrane glycoporins via ankyrin proteins (Skalak and Chien, 1982; Stokke et al., 1986). Membrane embedded proteins, including Band 3 proteins, and lipid microdomains have demonstrated to be important determinants erythrocyte membrane mechanics (Markin, 1981; Gimsa and Ried, 1995; Kralj-Iglič et al., 1996; Seifert, 1997; Huttner and Zimmerberg, 2001; McMahon and Gallop, 2005; Hägerstrand et al., 2006; Baumgart et al., 2011; Walani et al., 2014; Fosnaric et al., 2019). Additionally, the actin components are themselves atypical, consisting of short, uniform length filaments arranged in a highly irregular hexagonal lattice, with subpopulations of dynamic filaments highly dependent on the shear modulus and modulus of area compression (Mohandas and Evans, 1994; Gokhin et al., 2015). The spectrin-actin network itself is important for the stabilization of many erythrocyte shapes, including spiculated RBCs (Iglič, 1997; Iglič et al., 1998; Mukhopadhyay et al., 2002). Additionally, the non-linear viscoelastic mechanical properties of the RBC membrane results in context-dependent elastodynamic behavior that stems from changes in shear modulus (Stokke et al., 1986; Secomb and Hsu, 1995). In large vessels, RBCs aggregate together along the central streamline in Rouleaux-stack formations. As vessel diameter decreases and shear rate increases, these formations reversibly break down into flowing discocytes, which successively roll, tumble, and deform in areas proximal to the vessel cell free layer (Lanotte et al., 2016). Furthermore, increasing shear rate results in deformations of RBCs along streamlines, contributing to the non-Newtonian shear-thinning behavior of blood (Secomb, 1991; Baskurt and Meiselman, 2003). Within capillaries, RBCs undergo deformations unlike those in larger microvessels, adopting a non-axisymmetric parachute-like conformation, which minimizes the shear modulus (Secomb et al., 1983; Secomb and Hsu, 1995). The RBC membrane itself is not static and undergoes tank-treadmilling to compensate for the increase in fluid viscosity. While tank-treadmilling motion increases membrane energy dissipation, cytoplasmic energy dissipation is minimized, preventing hemolysis and capillary plugging (Lanotte et al., 2016). Together, these aspects of RBCs impart the ability for these cells to withstand large shear stresses in the larger vessels while being able to deform through capillaries with ease.

Changes in RBC deformability are characteristics of mechanical insult to RBC membranes in various hemoglobinopathies, vascular disease, and during storage lesion. In Sickle Cell Disease (SCD), HbS polymerization forces erythrocyte dependent shape changes, increasing membrane viscosity and membrane rigidity (Nash et al., 1984). These mechanical changes contribute to the pathogenesis of vaso-occlusive crises in patients with SCD, which remains a clinical problem (Messmann et al., 1990). Similar changes in mechanical properties and the mechanical stability of RBCs have been observed in symptomatic thalassemia (Schrier et al., 1989). Changes in RBC deformability have also been found to be significantly associated with the pathogeneis of microvascular stenosis and retinal vascular disorders (Agrawal et al., 2016; Vahidkhah et al., 2016). During storage lesion, irreversible changes to the RBC membrane result in decreased deformability, contributing to the increased hemolysis, microvascular injury, and decreased perfusion observed post transfusion. The pathogenesis of these donor-dependent mechanical changes during storage lesion results from oxidative damage to lipid membranes, which are the primary component of RBC membrane elasticity (Jani et al., 2017a,b). These pathologic changes in RBC mechanical properties reflect the need for the development of *in vivo* methods for quantification of RBC mechanical properties in the microvasculature.

The viscoelastic mechanical properties of the human RBC membrane, namely deformation, volume loss, and rupture, have been traditionally assessed via the method of micropipette aspiration (Hochmuth, 2000; Baskurt and Meiselman, 2003). Micropipette aspiration involves application of an external pressure on a local area of the membrane, resulting in membrane deformation. Quantification of this deformation allows for the determination of the membrane elastic modulus via the Law of Laplace. However, micropipette aspiration fails to account for the context-dependent mechanics of the RBC and the parachute-like morphology adopted by these cells in capillaries. More sophistical techniques, namely ektacyotmetry, allow for the determination of the mechanical response to shear stress (Parrow et al., 2018). Molecular techniques, namely atomic force microscopy and optical tweezers, allow for increased spatial resolution by determining spatial maps of stress along the RBC membrane (Fedosov et al., 2014). Recently, microfluidic devices have been developed for probing the mechanical properties of RBCs and the effects of mechanical stress on RBCs in hematologic conditions, namely sickle cell disease (Iragorri et al., 2018; Ye et al., 2019). The mechanical properties and the strain energy function for the RBC membrane have been defined for a two-dimensional membrane with variable membrane thickness, physiologically dependent on the density of glycoproteins and transporters at any one given section of the RBC membrane (Skalak and Chien, 1982). In these models, the bending moments are assumed to be isotropic and proportional to the gradient of curvature along the surface, neglecting internal stresses (Secomb, 1991; Secomb and Hsu, 1995). The aforementioned model, known as Skalak's Law, determines the path integral of stress along the membrane axis, or membrane tension, and predicts mechanics dependent on changes in the shear modulus (Mohandas and Evans, 1994). More sophisticated models have incorporated the gel like behavior of the RBC by quantifying cytoplasmic viscosity from hemoglobin (Stokke et al., 1986). Recent studies have incorporated the molecular basis for membrane deformation by creating finite element models for actin-spectrin interactions. These models have been used to support measurements of RBC mechanical properties *ex vivo* and simulate RBC dynamics during capillary plug flow (Stokke et al., 1986). Particle tracking and dissipative particle dynamics have recently been applied for diagnostic purposes by utilizing semi-continuum models using immersive boundary or front tracking techniques to exploit pathologic changes in RBC membrane rigidity that result from parasitic infection (Fedosov et al., 2014). To our knowledge, however, these models have been all applied *in vitro* and no methods have been designed to estimate RBC membrane mechanics and wall shear stress *in vivo* during capillary plug flow.

The objective of this study is to quantify the mechanical properties and wall shear stress experienced by RBC membranes during capillary plug flow *in vivo* utilizing intravital imaging and computational methods. Capillaries were imaged in the rat cremaster muscle pre- and post-RBC transfusion with 2-week stored cells, and mechanical properties, shear stress, and shear strain were quantified. Briefly, RBC membrane shapes were extracted utilizing image processing and a custom tracing and parametrization algorithm. RBC parameterizations were used to determine updated deformation gradient and Lagrangian-Green strain tensors for each point along the parametrization and for each frame during plug flow. The updated Lagrangian Green strain and Displacement Gradient tensors were numerically fit to the Navier-Lame equations along the parameterized boundary to determined Lame's constants. The RBC membrane itself is modeled as a two-dimensional Hookean Isotropic Solid, and the Cauchy stress tensor was determined utilized the Lagrangian Green strain tensor and the numerically determined Lame's constants. This study is the first to provide a comprehensive methodology for quantifying the deformation of RBCs *in vivo* without the need for physical tracking mechanisms such as fluorescent beads or tags.

## 2. Materials and Methods

### 2.1. Animal Model and Tissue Preparation

Animal handling and care procedures were in accordance with the National Institutes of Health Guide for the Care and Use of Laboratory Animals, 2011. The University of California, San Diego Animal Subjects Committee approved the experimental protocol. Twelve Sprague-Dawley rats weighing 140–190 g were used for intravital microscopy examination; additional animals were used as plasma and RBC donors. Anesthesia was induced using 60 mg/kg i.p of pentobarbital sodium (Nembutal, Lundbeck Inc, Deerfield, IL) and supplemented as needed by i.v. infusion of 20% of the initial dose. Animals were placed on a heating pad to maintain body temperature at 37°C. The jugular vein and femoral artery were catheterized, and a tracheal tube was inserted to support ventilation. Catheters were pre-filled with heparinized saline solution (30 IU/ml).

The rat cremaster muscle was exteriorized as previously described by Baez (1973). The rat was positioned on a Plexiglas plate, and the cremaster muscle was secured to a heated platform for viewing. The platform enabled muscle temperature maintenance at 35°C during surgery and the experiment. The muscle was moistened with warm Plasma-Lyte solution (≈ 37°C) and following surgical preparation, exposure to atmospheric oxygen was prevented by a covering of polyvinyl film (Saran, SC Johnson & Son, Racine, WI).

### 2.2. Intravital Microscopy

**Figure 1:**
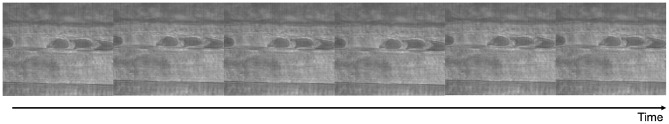
Capillary intravitral microscopy. Intravital microscopy of capillary plug flow *in vivo* in the rat cremaster muscle.

The experimental setup consisted of an intravital microscope (Olympus-BX51WI) equipped with a matching long working distance condenser (NA = 0.8, Thorlabs, Newton, NJ). Two magnifications of 2X and 1.5X were installed between the objective (60X, LUMPFL-WIR, NA = 0.8; Olympus) and the high-speed camera, providing a total magnification of 1800X. A mercury arc lamp (100 W, Walker Instruments, Scottsdale, AZ) was used to illuminate the tissue. A 400-nm interference filter (Spectra Physics, no. 59820) was placed above the condenser in the light path to maximize contrast between blood and the surrounding tissue. A high-speed video camera (Fastcam 1024 PCI, Photron USA), equipped with a one-megapixel chip was used for video recording. Additionally, all videos were recorded between 1,000 and 2,000 frames per second and the camera shutter speed was optimized to obtain the highest quality image possible. Videos were saved as 8-bit graysclae image stacks. A representative intravital microscopy time-lapse video is shown in Figure 1.

### 2.3. Blood and Plasma Collection

Whole blood was obtained from rat donors, adult male Sprague-Dawley rats (280–300 g, *n* = 24). Briefly, rats were anesthetized, left carotid artery catheter was implanted, and blood was allowed to flow into heparinized tubes (sodium heparin 15 IU/ml). RBCs and plasma were separated by centrifugation (2,700 rpm, 7 min). The buffy coat was discarded. RBCs morphology was checked prior to infusion and discarded if cells were crenated. Blood was stored for 2 weeks prior to transfusion.

### 2.4. Isovolumic Exchange Transfusion Protocol

To adjust the Hct_syst_ to the target Hct level the volume of the exchange-transfusion was calculated from the formula, exchange volume = 0.024 x ΔHct, where V is the equal to the rat's blood volume in ml (estimated as 6% of the weight in grams) and ΔHct is the difference between the initial and the target Hct (Cabrales et al., 2008). Packed RBCs or plasma were infused through the jugular vein catheter and an equal volume of blood was withdrawn from the femoral artery, simultaneously, at a rate of 0.3 ml/min to prevent rapid shifts in blood volume.

### 2.5. Systemic Parameters and Blood Measurements

Mean arterial pressure (MAP) and heart rate (HR) were recorded using a pressure transducer connected to the femoral artery catheter using a MP-150 (BIOPAC Systems, Goleta, CA). Blood samples were collected from the femoral artery catheter in heparinized capillary tubes (Fisher Scientific, Pittsburgh, PA) to determine Hctsyst by centrifugation (5 min at 8,000 rpm, Readacrit; Clay Adams, Division of Becton Dickinson, Parsipanny, NJ). Additionally, blood mixtures were prepared using packed RBCs and fresh plasma to determine blood and plasma viscosities (at 37°C and shear rates of between 50 and 450 s^−1^) using a cone-plate viscometer (DV II + Pro, Brookfield, Middleboro, MA, USA).

### 2.6. Experiment Protocol

Imaging for intravital microscopy of isolated capillaries with good image quality, and image contrast were selected. Blood sample and microcirculation videos were taken 10 min after the end of the 40% exchange transfusion. Video recording of these capillaries were taken over 1,632 frames (1,000–2,000 fps).

### 2.7. Cell Tracking and Image Processing

**Figure 2:**
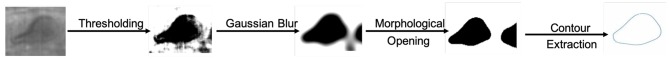
Image processing and contour extraction. Novel image processing were applied to obtain the erythrocyte contour for analysis. Briefly, image processing to obtain binary images involved the following: (1) contrast thresholding ([μ_*i*_ − σ_*i*_, μ_*i*_] → [0, 255], (2) Gaussian Smoothing (kernel: 5, σ = 7), (3) Thresholding (T = 68), (4) morphological opening (kernel: 3x3), and (5) binary edge detection for contour extraction.

RBCs were manually identified and tracked by the user utilizing a user interface developed in MATLAB (Mathworks, Natick, MA). Following cell selection and tracking, a region of interest containing the cell was generated, and image processing was performed to obtain the parametrization. Briefly, image processing to obtain binary images involved the following: (1) contrast thresholding ([μ_*i*_ − σ_*i*_, μ_*i*_] → [0, 255]), (2) Gaussian Smoothing (kernel: 5, σ = 7), (3) Thresholding (T = 68), and (4) morphological opening (kernel: 3x3). All image processing was performed in MATLAB (Mathworks, Natick, MA). Contour extraction involved edge detection of the processed binary images, obtained utilizing a MATLAB implementation of the Moore-Neighbor algorithm for binary image edge detection (Reddy et al., 2012). Following edge detection, parameterizations of edge pixel positions were obtained utilizing MATLAB (Mathworks, Natick, MA). Image transformations are expressed in detail in Figure 2.

#### 2.7.1. Contrast Stretching

Contrast enhancement was applied by mapping all the intensity values within a range [*a, b*], of an image *f*, to the full range of grayscale intensity values [0, 255] in a new image *g*. Defining the stretching function as *S*(*f*_*ij*_), the mapped range of the intensity values is determined as follows:

(2.1) $$\begin{array}{l} S\left(f_{ij}\right)=\left(f_{ij}-a\right)\frac{\left(b-a\right)}{255} \end{array}$$

(2.2) $$\begin{array}{l} g_{ij}=\{\begin{array}{cc} S\left(f_{ij}\right) & 0\leq S\left(f_{ij}\right)\leq 255\\ 0 & S\left(f_{ij}\right)<0\\ 255 & S\left(f_{ij}\right)>255 \end{array} \end{array}$$

The histogram of image *f* is assumed to be normally distributed with mean μ_*f*_ and standard deviation σ_*f*_. Optimal contrast enhancement was determined empirically as [μ_*f*_ − σ_*f*_, μ_*f*_] is mapped to [0, 255]. Results for contrast stretching are shown in Figure 2.

#### 2.7.2. Gaussian Smoothing

Gaussian smoothing was applied to obtain well-defined boundaries for the erythrocyte. A Gaussian Kernel *K* of size [*MxM*] was utilized, where

(2.3) $$\begin{array}{l} L_{ij}=G\left(x,y\right)=\frac{1}{2\pi \sigma^{2}}e^{-\frac{\left(x^{2}+y^{2}\right)}{2\sigma^{2}}} \end{array}$$

(2.4) $$\begin{array}{l} K_{kl}=\frac{1}{\sum_{i}\sum_{j}\left(L_{ij}\right)}L_{kl} \end{array}$$

Gaussian smoothing was performed by convolving the image, *f*, with the kernel *K*, *g* = *K* ∘ *f*.

#### 2.7.3. Binary Thresholding and Morphological Opening

Binary thresholding for edge detection involved application of a thresholding function *T*(*f*_*ij*_), as follows:

(2.5) $$\begin{array}{l} T\left(f_{ij}\right)=\{\begin{array}{cc} 1 & f_{ij}\geq \beta \\ 0 & f_{ij}<\beta \end{array} \end{array}$$

(2.6) $$\begin{array}{l} g_{ij}=T\left(f_{ij}\right) \end{array}$$

A threshold intensity of β = 68 was found to empirically yield the best results. Morphological opening was applied to thresholded binary images to eliminate overlapping boundaries between neighboring erythrocytes. Briefly, a structural element, *S*, is defined as a binary [*MxN*] matrix. The kernel is then moved over an image *f* and if *f*_*ij*_ = *s*_*ij*_ = 1 for all *i* ∈ [1, *M*] and *j* ∈ [1, *N*] then, then *S* is said to “fit” *f*. If *f*_*ij*_ = *S*_*ij*_ = 1 for some *i* ∈ [1, *M*] and *j* ∈ [1, *N*], then *S* is said to “hit” *f*.

A morphological erosion of an image *f*, defined as *g* = *f* ⊖ *s*, creates a new image *g* defined by:

(2.7) $$\begin{array}{l} g_{ij}=\{\begin{array}{cc} 1 & S“\text{fits}”P\\ 0 & otherwise \end{array} \end{array}$$

where *P* is defined as:

(2.8) $$\begin{array}{l} P_{kl}=f_{mn}\{\begin{array}{c} k\in \left[1,M\right]\\ l\in \left[1,N\right]\\ m\in \left[i-\frac{\left(M-1\right)}{2},i+\frac{\left(M-1\right)}{2}\right]\\ n\in \left[j-\frac{\left(N-1\right)}{2},j+\frac{\left(N-1\right)}{2}\right] \end{array} \end{array}$$

A morphological dilation of an image *f*, defined as *g* = *f* ⊕ *s*, creates a new image *g* defined by:

(2.9) $$\begin{array}{l} g_{ij}=\{\begin{array}{cc} 1 & S“\text{hits}”P\\ 0 & otherwise \end{array} \end{array}$$

where *P* is defined as:

(2.10) $$\begin{array}{l} P_{kl}=f_{mn}\{\begin{array}{c} k\in \left[1,M\right]\\ l\in \left[1,N\right]\\ m\in \left[i-\frac{\left(M-1\right)}{2},i+\frac{\left(M-1\right)}{2}\right]\\ n\in \left[j-\frac{\left(N-1\right)}{2},j+\frac{\left(N-1\right)}{2}\right] \end{array} \end{array}$$

The process of morphological opening to eliminate shared boundaries between neighboring cells is thus defined as:

(2.11) $$\begin{array}{l} g=S⊕\left(S⊖f\right) \end{array}$$

Morphological opening was applied to obtain isolate object boundaries for edge detection.

#### 2.7.4. Binary Edge Detection

**Figure 3:**
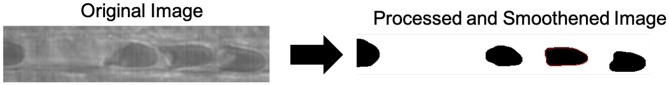
Cell tracking and contour extraction. A custom program for manual cell tracking was developed in MATLAB. Briefly, an image processing transformation was applied to an intravital microscopy time-lapse video of capillary plug flow, followed by binary edge detection and contour smoothing. The identified contour of the smoothed cell is observed in red.

Contour extraction involved edge detection of the processed binary images, obtained utilizing a MATLAB implementation of the Moore-Neighbor algorithm for binary image edge detection (Reddy et al., 2012). Erythrocytes were manually tracked utilizing MATLAB, and contours were extracted. Figure 3 demonstrates the erythrocyte tracking and contour extraction methods utilized.

### 2.8. Determination of the Deformation Gradient and Lagrangian Green Strain Tensor

A discretized deformation gradient tensor, **F**, was determined from the discrete parametrization by the method of Gullett et al. (2007) and Zimmerman et al. (2009). Briefly, the discrete deformation gradient tensor was defined by minimizing the error between clusters of L points weighted by a cubic spline function. In the case of this analysis, there are *N* deformed states (different time points) and *M* points that parametrize the edge, which is being analyzed. Therefore, there are a total of *M*(*N* − 1) deformation gradient tensors *F*_*iR*_. As a result, the *F*_*iR*_ is a function of the initial position *X*_*R*_ = (*X*_1_, *X*_2_) from a Lagrangian perspective.

(2.12) $$\begin{array}{l} F_{iR}=F_{iR}\left(X_{1},X_{2}\right) \end{array}$$

The deformation gradient tensor is therefore numerically determined between deformed states. Instead of referencing the deformation relative to the initial position, an updated Lagrangian methodology will be used, such that the deformation gradient tensor for all the points in a single frame, *F*_*iR*_ = *F*_*iR*_(*X*_1_, *X*_2_), is a function of the previous frame. Mathematically, for the *N*th frame, this is written as:

(2.13) $$\begin{array}{l} F_{iR}^{N}=f\left(F_{iR}^{N-1}\right) \end{array}$$

Let the coordinate system of the (*N* − 1)th frame be denoted as: $X_{R}^{N-1}=\left(X_{1}^{N-1},X_{2}^{N-1}\right)$. The deformation gradient tensor of the *N*th frame is therefore a function of the coordinate system of the (*N* − 1)th frame:

(2.14) $$\begin{array}{l} F_{iR}^{N}=F_{iR}^{N}\left(X_{1}^{N-1},X_{2}^{N-1}\right) \end{array}$$

Using the formulation defined by Gullett et al., a discrete deformation gradient tensor *F*_*iR*_ can be defined. The discrete deformation gradient tensor can be defined by minimizing the error in the mapping between clusters of *L* points. Let Δ*X*^*il*^ represent the change in the vector $\vec{X}$ in the *i*th point in the *j*th frame relative to the *l*th point in the cluster of *L* points of the undeformed state, and Δ*x*^*il*^ represent the change in the vector $\vec{x}$ in the *i*th point in the *j*th frame relative to the *l*th point in the cluster of *L* points of the deformed state. The discretized deformation gradient tensor $\hat{F_{iR}}$ can be calculated as:

(2.15) $$\begin{array}{l} \hat{F_{ab}}\underset{M}{\sum }\Delta X_{b}^{il}\Delta X_{j}^{il}w_{M}=\underset{M}{\sum }\Delta x_{a}^{il}\Delta X_{j}^{il}w_{M} \end{array}$$

In matrix notation, this is:

(2.16) $$\begin{array}{l} \hat{\text{F}}\underset{M}{\sum }\Delta X^{il}\Delta X^{il^{T}}w_{M}=\underset{M}{\sum }\Delta x^{il}\Delta X^{il^{T}}w_{M} \end{array}$$

Therefore, the deformation gradient tensor, $\hat{F}$ is:

(2.17) $$\begin{array}{l} \hat{\text{F}}=AD^{-1} \end{array}$$

where

(2.18) $$\begin{array}{l} A=\underset{M}{\sum }\Delta x^{il}\Delta X^{il^{T}}w_{M} \end{array}$$

and

(2.19) $$\begin{array}{l} D=\underset{M}{\sum }\Delta X^{il}\Delta X^{il^{T}}w_{M} \end{array}$$

where *w*_*M*_ is a weighting function for all M points, giving more weight to the *i*th point in the cluster of *L* points. A cubic spline was used for the weighting function *w*(*r*):

(2.20) $$\begin{array}{l} w\left(r\right)=\{\begin{array}{cc} 1-6r^{2}+6r^{3} & r\leq \frac{1}{2}\\ 2-6r+6r^{2}-2r^{3} & \frac{1}{2}<r<1.0\\ 0 & r\geq 1.0 \end{array} \end{array}$$

where *r* is the adjusted radius of the cluster defined as:

(2.21) $$\begin{array}{l} r=\frac{r_{gl}-r_{g1}}{r_{cut}} \end{array}$$

where *r*_*g*1_ and *r*_*gl*_ are the distances between the current point and the first and *l*th points respectively, and *r*_*cut*_ is the cutoff radius, which is the maximum distance between the reference point and any point in the cluster. A large cutoff radius was utilized to account for membrane treadmilling during erythrocyte deformation. Furthermore, to account for discontinuities from image processing at horizontal and vertical edges, posts near this vicinity were ignored.

The Cauchy strain tensor was determined assuming the infinitesimal strain assumption from the deformation gradients:

(2.22) $$\begin{array}{l} \epsilon =\frac{1}{2}\left(\text{F}+{\text{F}}^{T}\right)-\text{I} \end{array}$$

To adjust for membrane viscoelasticity, the membrane was treated as a Maxwell solid, such that the elastic strain was an empiric fraction β of the total strain, where β = 0.1. Due to the short time scale, effects of shape memory are neglected. As consistent with the Maxwell model, we assume only shape memory during the time of imaging. To adjust for membrane geometry, the strain tensor was multiplied by a ratio of the membrane area, with constant thickness *w*_*n*_ = 5 nm, and total cell area, determined from image processing:

(2.23) $$\begin{array}{l} \epsilon =\frac{\beta w_{n}l}{A}\left(\frac{1}{2}\left(\text{F}+{\text{F}}^{T}\right)-\text{I}\right) \end{array}$$

where *A* is total cell area and *l* is total cell perimeter.

### 2.9. Determination of Body Forces

**Figure 4:**
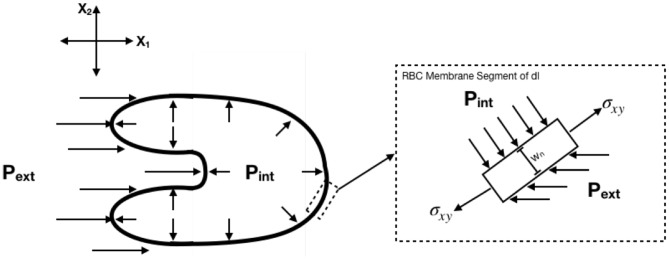
Free body diagram for determination of body forces. Body forces were calculated assuming constant internal pressure and external pressure determined as a function of time.

Body forces were determined with the following assumptions: (1) Each RBC is a thin, 2D membrane with constant thickness *w*_*n*_ = 5 nm, (2) Internal stresses are replaced by a single constant internal pressure *P*_int_ = 85 dynes/cm^2^ acting perpendicular to the membrane (Tran-Son-Tay et al., 1987), (3) The external pressure gradient is replaced by a single horizontal acting pressure acting left of the center of area, *P*_ext_. All calculations were performed in Lagrangian coordinates such that the cell center of area was defined as the origin. The free body diagram for the system is shown in Figure 4. Expressions for body forces from the free body diagram were determined to be the following:

(2.24) $$\begin{array}{l} \rho {\text{b}}_{X}=\langle -\frac{1}{w_{n}}\left(P_{ext}+P_{int}cos\theta \right),-\frac{1}{w_{n}}P_{int}sin\theta \rangle \end{array}$$

where ρ is membrane density, approximated as 1,300 kg/m^3^ (Tran-Son-Tay et al., 1987). The relative external pressure for an RBC flowing in a capillary was adopted from the method developed by Fung et al., assuming that capillary diameter is approximately equal to that of RBC diameter (Fung and Zweifach, 1971). In this model, arterial pressure is replaced by the arterial-venous pressure gradient, allowing for a spatial external pressure function.

(2.25) $$\begin{array}{l} P_{ext}\left(t\right)\\ =\{\begin{array}{cc} \frac{23\mu \left(V_{C}\left(t\right)+\alpha \left(t\right)\right)}{D_{T}}\frac{L_{T}}{D_{T}}(1-\frac{D_{T}}{L_{T}}\frac{0.8V_{C}\left(t\right)}{V_{C}\left(t\right)+\alpha \left(t\right)}) & X_{1}\leq 0\\ 0 & X_{1}>0 \end{array} \end{array}$$

where μ is blood viscosity (2.63 cP), *V*_*c*_ is capillary velocity, determined by pixels traveled by a cell in *t* frame, *L*_*t*_ is the length of the capillary, *D*_*t*_ is the diameter of the capillary, and α is the RBC displacement shift relative to plasma.

### 2.10. Determination of Mechanical Constants

From the Navier-Cauchy Equations of Elastodynamics:

(2.26) $$\begin{array}{l} \left(\lambda +\mu \right)\nabla \left(\nabla \cdot \vec{u}\right)+\mu \nabla^{2}\vec{u}+\rho \vec{b}=\rho \frac{\partial^{2}\vec{u}}{\partial t^{2}} \end{array}$$

where $\vec{u}=\langle u\left(X_{1},X_{2}\right),v\left(X_{1},X_{2}\right)\rangle$ is the vector of displacements in the *X*_1_ and *X*_2_ directions, ρ is the average membrane density, approximated as 1,300 kg/m^2^, $\vec{b}$ are the body forces acting on the membrane, and λ and μ are Lamé's First and Second parameters, respectively (Tran-Son-Tay et al., 1987). Multiplying the equation by *dA* = *w*_*n*_*dl*, which is the area of the infinitesimal piece of membrane for which these equations must hold yields:

(2.27) $$\begin{array}{l} \left(\lambda +\mu \right)\nabla \left(\nabla \cdot \vec{u}\right)dA+\mu \nabla^{2}\vec{u}dA+\rho \vec{b}dA=\rho \frac{\partial^{2}\vec{u}}{\partial t^{2}}dA \end{array}$$

When this occurs, the body forces simplify to the following expressions:

(2.28) $$\begin{array}{l} \rho b_{X_{1}}dA=-\left(P_{ext}+P_{int}cos\theta \right)dl \end{array}$$

(2.29) $$\begin{array}{l} \rho b_{X_{2}}dA=-w_{n}P_{int}sin\theta dl \end{array}$$

Dividing by *w*_*n*_ yields the following simplification of the Navier-Cauchy equations:

(2.30) $$\begin{array}{l} \left(\lambda +\mu \right)\nabla \left(\nabla \cdot \vec{u}\right)dl+\mu \nabla^{2}\vec{u}dl\\ \;-\frac{dl}{w_{n}}\langle P_{ext}+P_{int}cos\theta ,P_{int}sin\theta \rangle =\rho \frac{\partial^{2}\vec{u}}{\partial t^{2}}dl \end{array}$$

where *dl* can be approximated as:

(2.31) $$\begin{array}{l} dl\approx \frac{l}{M} \end{array}$$

where *M* is the number of points in a frame, which is constant between frames, and *l* is the perimeter of the RBC in a single frame.

Written out for a problem in two dimensions, these equations simplify to:

(2.32) $$\begin{array}{l} \left(\lambda +\mu \right)\left(\frac{\partial^{2}u}{\partial X_{1}^{2}}+\frac{\partial^{2}v}{\partial X_{1}\partial X_{2}}\right)dl+\mu \left(\frac{\partial^{2}u}{\partial X_{1}^{2}}+\frac{\partial^{2}u}{\partial X_{2}^{2}}\right)dl\\ \;-\frac{dl}{w_{n}}\left(P_{ext}+P_{int}cos\theta \right)=\rho \frac{\partial^{2}u}{\partial t^{2}}dl \end{array}$$

(2.33) $$\begin{array}{l} \left(\lambda +\mu \right)\left(\frac{\partial^{2}u}{\partial X_{2}\partial X_{1}}+\frac{\partial^{2}v}{\partial X_{2}^{2}}\right)dl+\mu \left(\frac{\partial^{2}v}{\partial X_{1}^{2}}+\frac{\partial^{2}v}{\partial X_{2}^{2}}\right)dl\\ \;-\frac{dl}{w_{n}}P_{int}sin\theta =\rho \frac{\partial^{2}v}{\partial t^{2}}dl \end{array}$$

When this is rewritten as a function of the components of the displacement gradient tensor, as well as the body forces $\vec{b}$, the equations simplify to:

(2.34) $$\begin{array}{l} \left(\lambda +2\mu \right)\frac{\partial G_{11}}{\partial X_{1}}dl+\left(\lambda +\mu \right)\frac{\partial G_{22}}{\partial X_{1}}dl+\mu \frac{\partial G_{12}}{\partial X_{2}}dl\\ \;-\frac{dl}{w_{n}}\left(P_{ext}+P_{int}cos\theta \right)=\rho \frac{\partial^{2}u}{\partial t^{2}}dl \end{array}$$

(2.35) $$\begin{array}{l} \left(\lambda +2\mu \right)\frac{\partial G_{22}}{\partial X_{2}}dl+\left(\lambda +\mu \right)\frac{\partial G_{11}}{\partial X_{2}}dl+\mu \frac{\partial G_{21}}{\partial X_{1}}dl\\ \;-\frac{dl}{w_{n}}P_{int}sin\theta =\rho \frac{\partial^{2}v}{\partial t^{2}}dl \end{array}$$

Using Cauchy boundary conditions, as the values of $\vec{u}=\langle u\left(X_{1},X_{2}\right),v\left(X_{1},X_{2}\right)\rangle$ are known along the boundary of the membrane at all time points (i.e., frames), as well as the fact that for any of the *j* of *N* frames, the boundary condition $X_{2}^{j}=f_{j}\left(X_{1}^{j}\right)$ must hold along the membrane, the boundary value problem numerically simplifies to a series of 2(*N* − 1)*M* equations, where the index *i* corresponds with one of the *M* points, and the index *j* corresponds with one of the *N* frames. Let $G_{RS}^{j}\left(X_{1,i},X_{2,i}\right)$ denote the displacement gradient tensor *G*_*RS*_ at point *i* in frame *j*. The boundary value problem can thus be solved at all *M* points and *N* frames under Cauchy boundary conditions via second order numerical difference approximations.

In matrix form, these equations are written as $A\vec{x}=\vec{b}$, where *A* is the matrix of convective forces $\vec{x}$ is the vector of Lamé's Constants, $\left[\begin{array}{l} \lambda \\ \mu \end{array}\right]$, and $\vec{b}$ is the vector of the sum of the inertial and body forces. Utilizing Equations (2.34) and (2.35), the matrix form can be written as:

(2.36) $$\begin{array}{l} dl\left[\begin{array}{cc} \frac{\partial G_{11}}{\partial X_{1}}+\frac{\partial G_{22}}{\partial X_{1}} & 2\frac{\partial G_{11}}{\partial X_{1}}+\frac{\partial G_{22}}{\partial X_{1}}+\frac{\partial G_{12}}{\partial X_{2}}\\ \frac{\partial G_{22}}{\partial X_{2}}+\frac{\partial G_{11}}{\partial X_{2}} & 2\frac{\partial G_{22}}{\partial X_{2}}+\frac{\partial G_{11}}{\partial X_{2}}+\frac{\partial G_{21}}{\partial X_{1}} \end{array}\right]\left[\begin{array}{c} \lambda \\ \mu \end{array}\right]\\ \;=\left[\begin{array}{c} \rho \frac{\partial^{2}u}{\partial t^{2}}dl+\frac{dl}{w_{n}}\left(P_{ext}+P_{int}cos\theta \right)\\ \rho \frac{\partial^{2}v}{\partial t^{2}}dl+\frac{dl}{w_{n}}P_{int}sin\theta \end{array}\right] \end{array}$$

Taking the Moore-Penrose pseudoinverse of the convective forces yields a numerical approximation for Lame's constants:

(2.37) $$\begin{array}{l} \left[\begin{array}{c} \lambda \\ \mu \end{array}\right]=\frac{1}{dl}\left[\begin{array}{c} A^{+} \end{array}\right]\left[\begin{array}{c} \rho \frac{\partial^{2}u}{\partial t^{2}}dl+\frac{dl}{w_{n}}\left(P_{ext}+P_{int}cos\theta \right)\\ \rho \frac{\partial^{2}v}{\partial t^{2}}dl+\frac{dl}{w_{n}}P_{int}sin\theta \end{array}\right] \end{array}$$

where [*A*^+^] isthe Moore-Penrose pseudoinverse of the matrix *A*, which is solved at every point, and averaged over all points to obtain a solution for a mean value for λ and μ.

### 2.11. Determination of Wall Shear Stress and Membrane Tension

The updated Cauchy stress tensor was determined utilizing Hooke's Law, assuming the RBC membrane to be a 2D Hookean Isotropic Solid.

(2.38) $$\begin{array}{l} {\text{T}}_{\text{ij}}=\lambda {\epsilon_{\text{aa}}}^{k}\delta_{ij}+2\mu {\epsilon_{\text{ij}}}^{k} \end{array}$$

where ϵ_**ij**_^k^ is the updated Lagrangian Green strain tensor for the *k*th frame. Wall shear stress, τ_1_ is therefore geometrically determined as:

(2.39) $$\begin{array}{l} \tau_{1}=T_{12}\cos\theta -\left(T_{11}-P_{\text{ext}}\right)\sin\theta \end{array}$$

To account for the assumed constant membrane thickness, the shear component of membrane tension, *t*_1_, was approximated by integrating wall shear stress over the thickness of the membrane:

(2.40) $$\begin{array}{l} t_{1}=\int_{0}^{w_{n}}\tau_{1}dw\approx \tau_{1}w_{n} \end{array}$$

### 2.12. Principle Component Analysis

Upon a 40% exchange transfusion with 2-week stored RBCs results, a heterogeneous population of RBCs was present in the circulation. Principle component analysis (PCA) was performed on vectors containing the 2 Lame constants, the Young's modulus, Bulk modulus, shear stress and shear strain, for each cell. The two principle components with the largest variance were separated, for each cell, in transfusion videos, and compared with a PCA model developed from fresh cell videos, used as a baseline. Principle components greater than 3 standard deviations from the mean baseline principle components were treated as a separate population for all calculations. All PCA was performed utilizing MATLAB (Mathworks, Natick, MA).

### 2.13. Statistical Analysis

Unless otherwise noted, variables are represented as mean ± standard error of the mean (SEM). All statistical calculations were performed with Prism 8 (GraphPad Software, San Diego, CA) and/or MATLAB (Mathworks, Natick, MA). SEM for mechanical properties, shear stress, and shear strain were calculated from the total number of erythrocytes used for analysis. Comparisons between mechanical constants, wall shear stress, and wall shear strain were performed using a student's *t*-test. Following principle component analysis, comparisons between mechanical constants, wall shear stress, and wall shear strain were performed using a one-way analysis of variance (ANOVA). *Post-hoc* analyses were performed using the Newman Keuls multiple comparison test. For all statistical tests, *P* < 0.01 was accepted as statistically significant. All animals passed the Grubbs test, ensuring that all the measured values at baseline were within a similar population (*P* < 0.01).

## 3. Results

A total of seven (*n* = 7) animals were entered into the study; two or three capillaries were selected for each animal after application of systemic and vessel reactivity inclusion criteria. Data from sixty-seven (*n* = 67; 32 in the before transfusion and 35 after transfusion were included in the results. There were no significant differences in hematocrit, systemic, or microhemodynamic parameters at baseline among animals.

### 3.1. Principle Component Analysis

**Figure 5:**
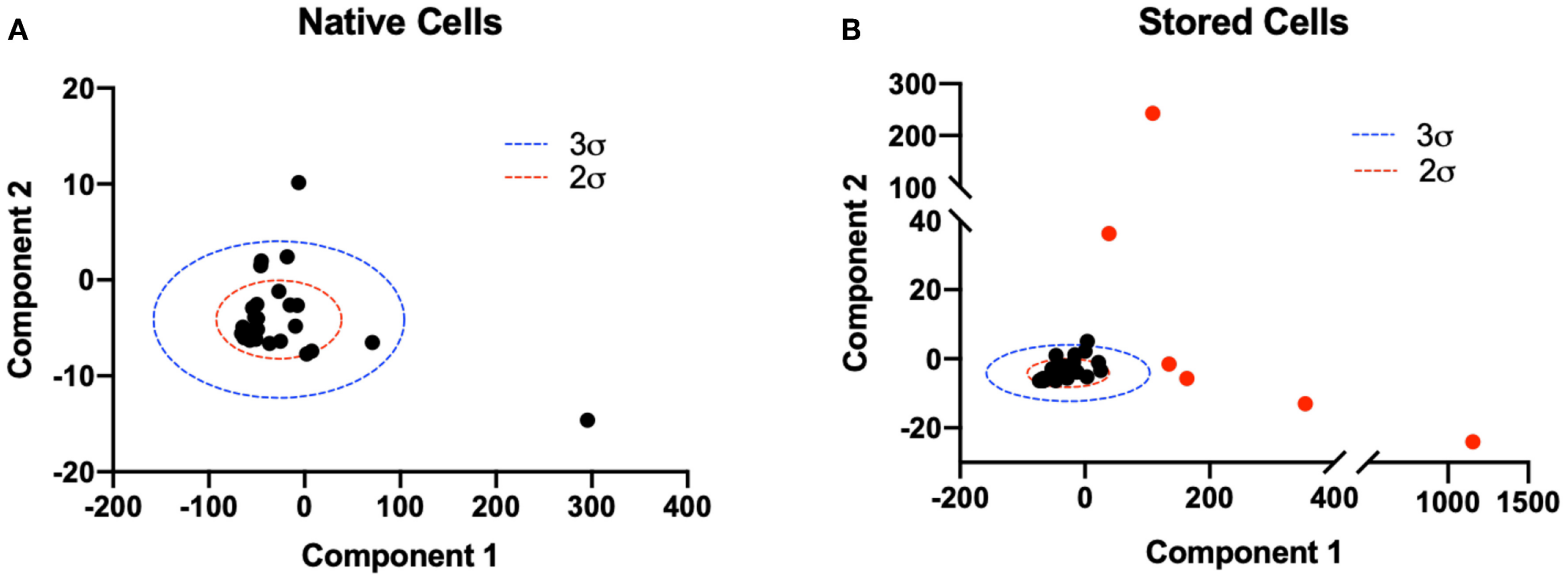
Principle component analysis of native and stored cell mechanical properties post transfusion. Principle component analysis of 6 numerically determined mechanical property variables was utilized to distinguish populations of cells post transfusion. The two principle components with largest variance were considered for analysis. **(A)** A two component PCA model was constructed for the population of native RBCs (*n* = 32). Two (red) and three (blue) standard deviations from the mean principle component vector are shown. **(B)** Two component PCA was performed on the population of erythrocytes analyzed in animals transfused with stored cells. Two (red) and three (blue) standard deviations from the mean principle component vector of native RBCs are plotted for reference. RBCs with both principle components greater than 3 standard deviations from the mean principle component of native RBCs were considered a separate population and are shown in red. Approximately 11/35 cells were found to satisfy this criteria.

Principle component analysis was utilized to identify and separate heterogeneous populations of erythro- cytes introduced in the experimental group (40% exchange transfusion). The two principle components with the largest variance were isolated from the population (*n* = 32) of native RBCs at baseline to construct a model to identify RBCs native to the circulation post transfusion (Figure 5A). In the population of erythrocytes analyzed from animals transfused with stored cells, RBCs with both principle components greater than 3 standard deviations from the mean principle component of native RBCs were identified and analyzed as a separate population for all calculations (Figure 5B). 68.6% (24/35) of RBCs analyzed from animals subjected to exchange transfusion were found to have both components within 3 standard deviations of the native RBC principle components (SC1). Additionally, 31.4% (11/35) of RBCs analyzed from animals subjected to exchange transfusion were found to have components greater than 3 standard deviations of the native RBC principle components (SC2). These results reflect the relative proportions of erythrocytes in these two populations expected after a 40% exchange transfusion.

### 3.2. Erythrocyte Mechanical Properties

**Figure 6:**
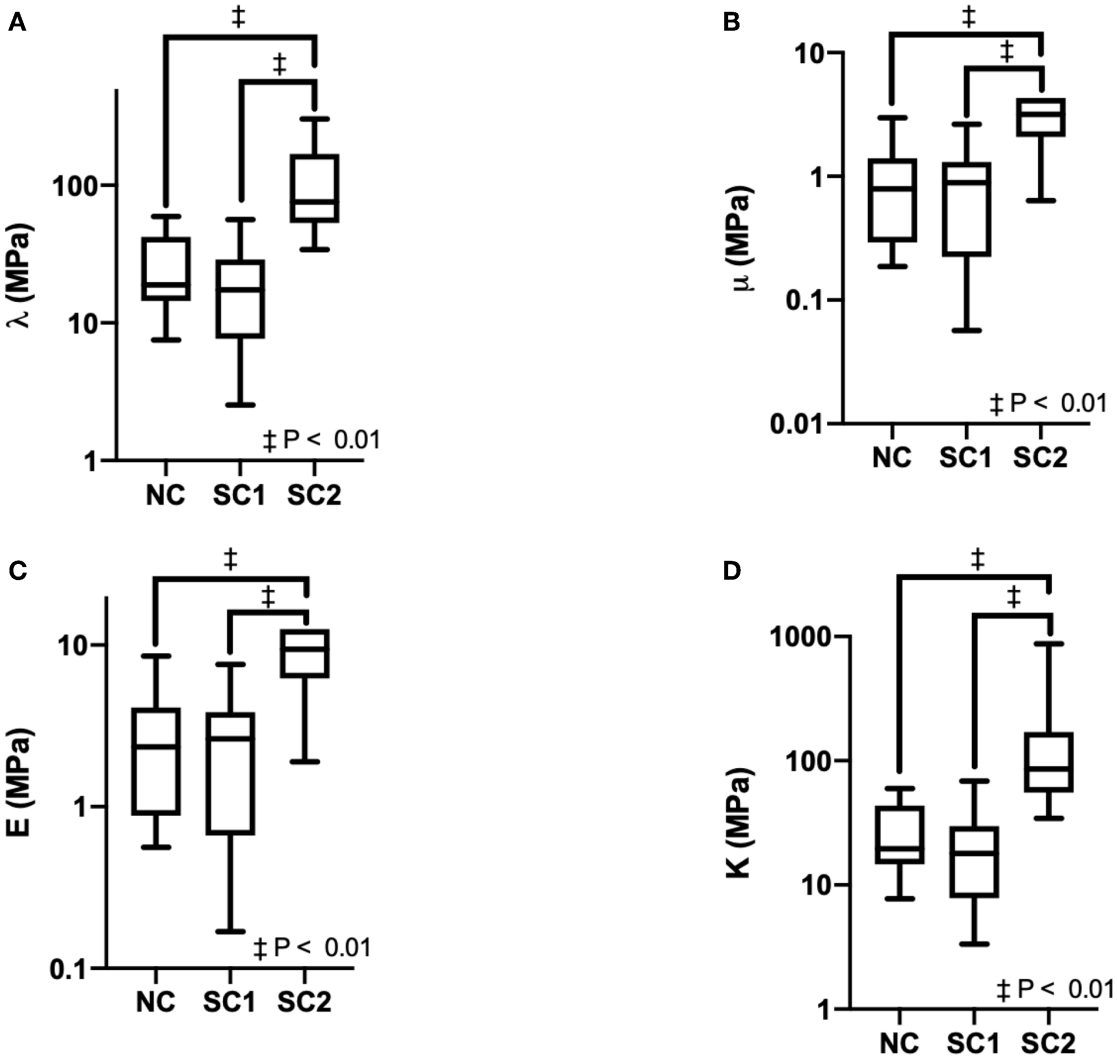
Erythrocyte mechanical properties. Erythrocyte mechanical properties were determined for Native Cells (NC), RBCs analyzed from animals with 40% exchange transfusion with principle components within 3 standard deviations of the control RBC principle components (SC1), and RBCs analyzed from animals with 40% exchange transfusion with principle components greater than 3 standard deviations of the control RBC principle components (SC2). The following mechanical constants were determined assuming Istotropic Hookean dynamics: **(A)** Lame's first constant (λ), **(B)** Shear Modulus (μ), **(C)** Young's Modulus (E), **(D)** Bulk Modulus (K).

Erythrocyte mechanical properties were determined via numerical methods utilizing the discretized displacement gradient tensor across the erythrocyte membrane over time and the Navier-Lame equations of elastodynamics. Results for Lame's first constant, Shear Modulus, Bulk Modulus, and Young's Modulus are summarized in Figure 6. Average Lame's first constant was calculated as 35 ± 8, 22 ± 4, and 170 ± 74 MPa for native RBCs (NC), SC1, and SC2, respectively. Similarly, average shear modulus was calculated as 1.1 ± 0.2, 0.90 ± 0.15, and 12 ± 8 MPa for NC, SC1, and SC2, respectively. Average young's modulus was calculated as 3.3 ± 0.6, 2.6 ± 0.4, and 32 ± 20 MPa for FC, SC1, and SC2, respectively. Average bulk modulus was calculated as 36 ± 8, 23 ± 4, and 180 ± 73 MPa for NC, SC1, and SC2, respectively. Lame's first constant, Shear Modulus, Bulk Modulus, and Young's Modulus were found to be statistically significantly higher (*P* < 0.01) for erythrocytes in population SC2 compared to NC. No statistically significant differences were observed for erythrocytes in population SC1 compared to NC, as expected. Together, these results suggest erythrocytes in population SC2 are less compliant and less deformable compared to NC.

### 3.3. Erythrocyte Wall Shear Stress and Shear Strain

**Figure 7:**
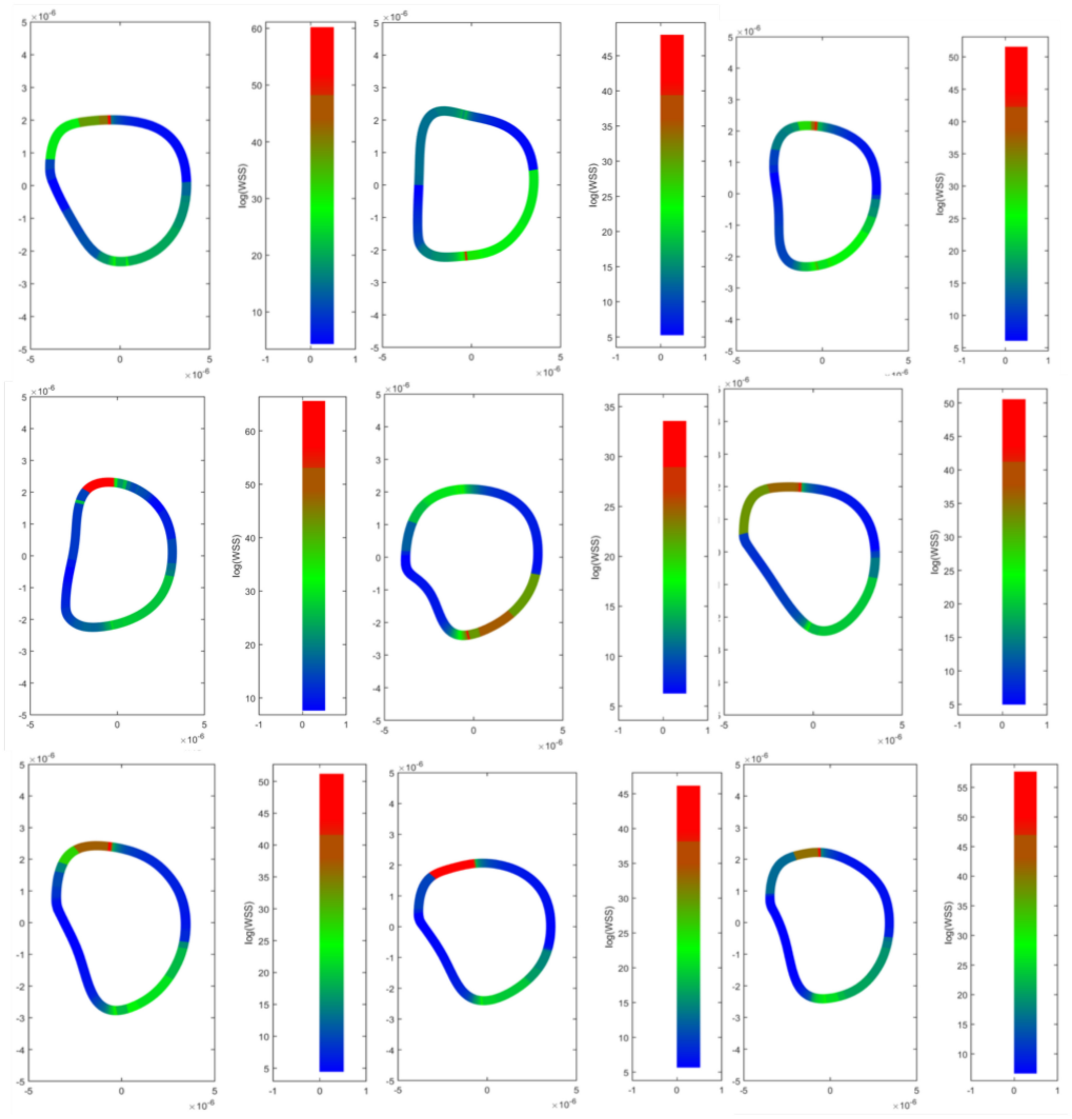
Wall shear stress distributions across erythrocyte membranes. Temporal averages of wall shear stress were visualized across the cell membrane for 9 different RBCs. Top Row–native RBCs (NC). Middle Row–RBCs from the SC1 population. Bottom Row–RBCs from the SC2 population. Stress concentrators were observed at contact sites between the RBC and the capillary endothelium despite not being specified in the model.

**Figure 8:**
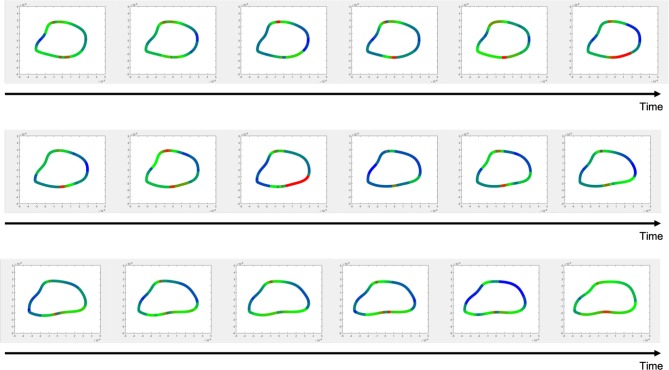
Time-lapsed wall shear stress distribution for a single erythrocyte.

The Cauchy stress tensor was determined for every pixel along the erythrocyte membrane over time. Shear stress was estimated from the stress tensor and averaged over time. Spatial maps of temporal average wall shear stress along the erythrocyte membrane are shown in Figure 7. A representative time lapse of a single erythrocyte is shown in Figure 8. Stress concentrators were observed at contact sites between the RBC and the capillary endothelium despite not being specified in the model.

**Figure 9:**
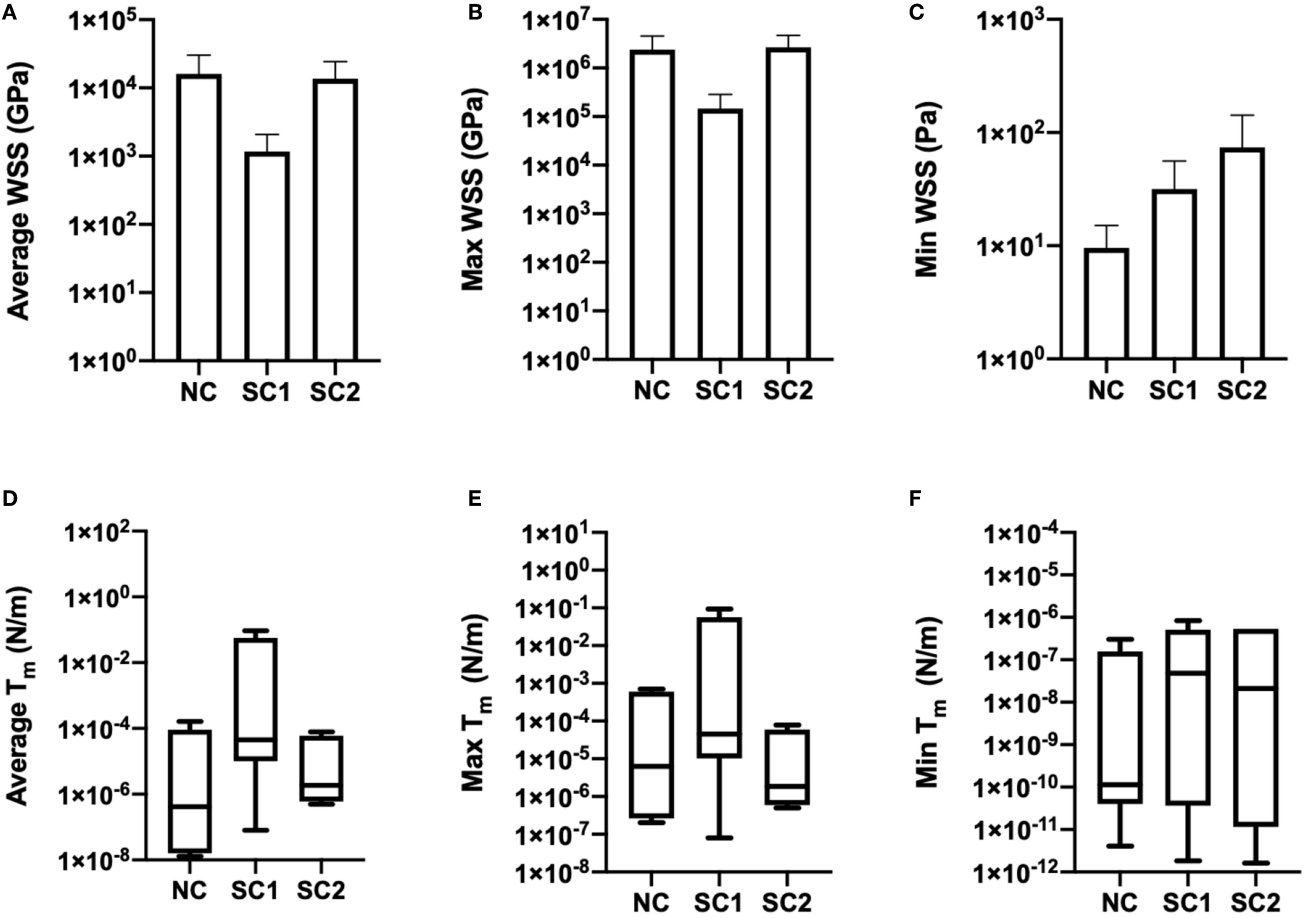
Wall shear stress and membrane tension. Temporal averages of WSS distributions were quantified and integrated over membrane thickness to determine membrane tension for native (NC), RBCs analyzed from animals with 40% exchange transfusion with principle components within 3 standard deviations of the control RBC principle components (SC1), and RBCs analyzed from animals with 40% exchange transfusion with principle components greater than 3 standard deviations of the control RBC principle components (SC2). **(A)** Average, **(B)** Maximum, and **(C)** Minimum Wall shear stress were not found be statistically different across the three populations of erythrocytes investigated. Furthermore, **(D)** Average, **(E)** Maximum, and **(F)** Minimum membrane tension were not observed to statistically different across the three populations of erythrocytes investigated.

**Figure 10:**
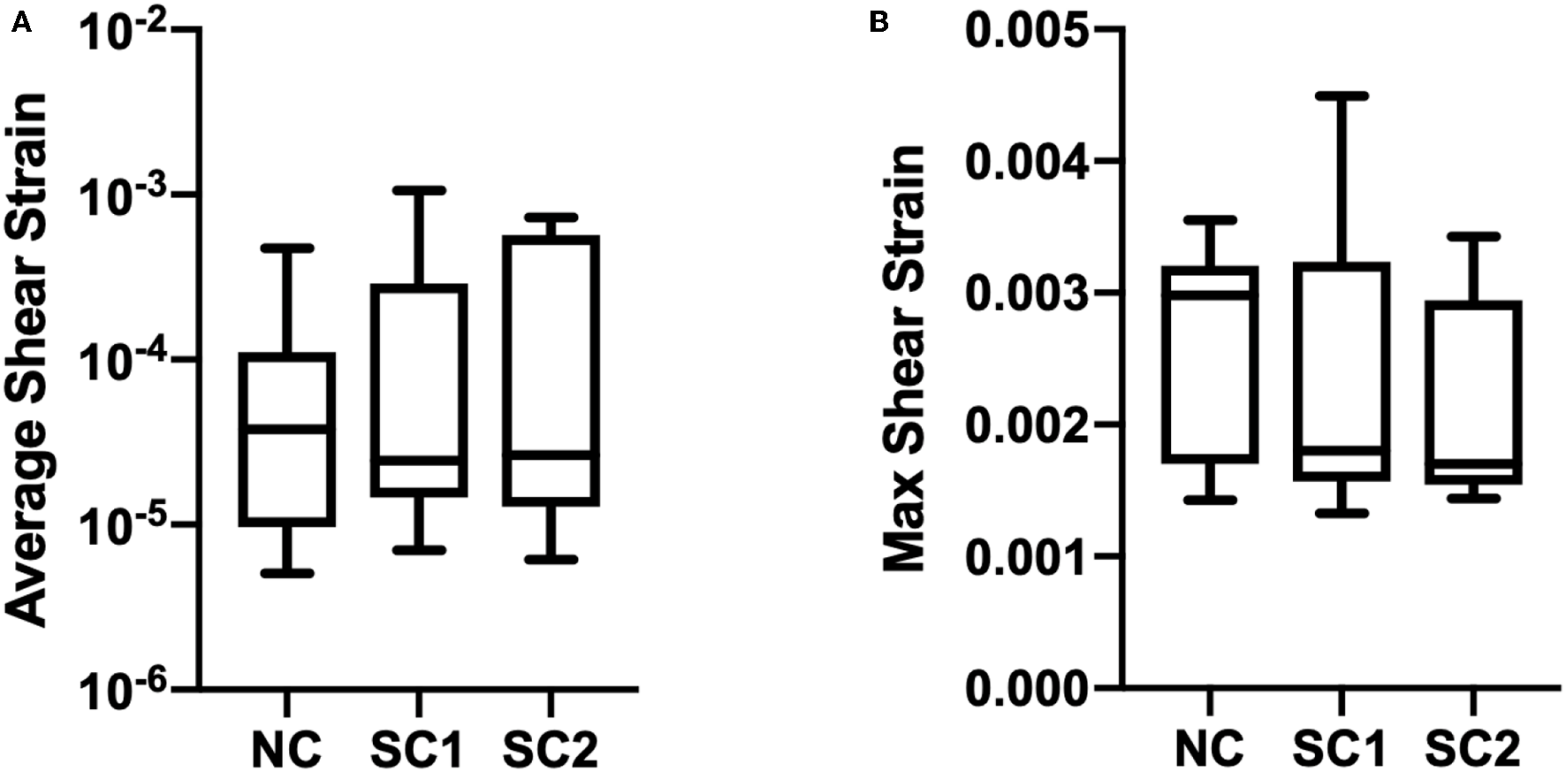
Erythrocyte wall shear strain. The updated Lagrangian Green strain tensor was quantified and temporally averaged for native (NC), RBCs analyzed from animals with 40% exchange transfusion with principle components within 3 standard deviations of the control RBC principle components (SC1), and RBCs analyzed from animals with 40% exchange transfusion with principle components greater than 3 standard deviations of the control RBC principle components (SC2). No significant differences were observed in **(A)** Average and **(B)** Maximum shear strain across the three populations of eyrthrocytes investigated.

Average, maximum, and minimum wall shear stress and membrane tension were quantified and are shown in Figure 9. In these results, maximum shear stress at contact sites between the RBC and the capillary endothelium is suggestive of the von Mises stress criterion of the erythrocyte during capillary plug flow. No statistically significant differences were observed for the average, maximum, and minimum wall shear stress and membrane tension across the three populations of erythrocytes investigated. Furthermore, no statistically significant differences were observed in average shear strain and maximum shear strain in the three populations of erythrocytes investigated (Figure 10). As expected, minimum shear strain was observed to be 0 at points of only tensile stresses for all erythrocytes investigated. Together, these results suggest that each of the cells were subjected to a similar mechanical environment and forces during capillary plug flow despite changes in membrane deformability.

## 4. Discussion

This study proposes a methodology that allows for the determination of the mechanical properties of RBCs in an *in vivo* setting, using a purely image processing-based approach. While an attempt was successfully made in order to get absolute quantities capable of characterizing the mechanical properties of RBCs, the main benefit of this approach is its ability to determine differences between distinct groups of RBCs present in the circulation. Furthermore, this approach is also novel in the sense that it does not require the use of physical markers, such as fluorescent beads or tags, in order to determine the deformation of the RBC membrane. This allows to analyze the deformations in the RBC membrane in a highly relevant physiological environment.

This study demonstrates a novel method for the quantification of erythrocyte mechanical properties and shear stress *in vivo* utilizing image processing and computational methods. Furthermore, application of PCA to the computationally determined shear stress, shear strain, and mechanical properties, allowed for the identification of heterogeneous populations of RBCs with statistically significant changes in mechanical properties after transfusion. These results indicate that both native erythrocytes and heterogeneous populations of erythrocytes identified by PCA were subjected to similar mechanical environment and forces during capillary plug flow despite the observed decrease in membrane deformability. Consequently, these results indicate that changes to perfusion and oxygen delivery observed by altering erythrocyte mechanical properties may result solely from changes in membrane deformability and physiologic processes important for its regulation, including the reticuloendothelial system. Furthermore, to our knowledge, this is the first study to quantify erythrocyte mechanics and membranes stresses *in vivo* during capillary plug flow utilizing measured strains. The mechanical properties and membrane stresses quantified from this study may therefore more accurately reflect shear dependent erythrocyte mechanics observed *in vivo*. The methods introduced in this study may provide a new avenue of investigation of erythrocyte mechanics in the context of hematologic conditions that adversely affect erythrocyte mechanical properties.

Principle component analysis allowed for the separation and identification of a heterogeneous population of RBCs in the experimental group. Further quantification of the mechanical properties of these heterogeneous RBCs demonstrated decreased compliance. We hypothesize that the erythrocytes with decreased compliance are those that were transfused post storage. It is well-known that RBCs undergo time dependent changes that impair deformability and various biochemical properties, namely pH, ATP, and 2,3 DPG concentration, known as storage lesion. While decay in biochemical properties is uniform across multiple populations of RBCs, our group has recently demonstrated that changes in deformability are variable and donor dependent (Jani et al., 2017a). As lipids are the most compliant of biomolecules, an increase of lipid oxidation injury and subsequent increase in membrane cell density during storage result in decreased compliance. Studies have also suggested that oxidative damage during storage is a pH dependent process and dependent on extracellular chloride concentration during storage, as the chloride-bicarbonate transporter is critical for the maintenance of intracellular pH (Donadee et al., 2011; Kim-Shapiro et al., 2011). Together, changes in pH and membrane composition seem to be the primary mediators of the decrease in deformability observed during storage lesion. To our knowledge, our study is the first to demonstrate, *in vivo* a heterogeneous population of erythrocytes post transfusion, suggesting that the observed impaired oxygen delivery and perfusion may be dependent on these mechanical changes, further demonstrating the wide applicability of this method.

Various methods have been utilized to quantify erythrocyte mechanical properties and membrane stresses due to the increasing importance of erythrocyte mechanics in the context of hematologic conditions. Of these techniques, micropipette aspiration is the most established and widely accepted (Hochmuth, 2000). Rand et al. reported an elastic modulus in the range of 0.7 to 3 MPa for erythrocytes measured via micropipette aspiration (Rand and Burton, 1964). These results, however, did not account for changes in elastic modulus in response to shear flow observed *in vivo*. Attempts by Hocmuth et al. to measure flow dependent elastic modulus utilizing a model cell tethered to the surface of a Couette flow channel reported an elastic modulus in the same range, ~1 MPa (Hochmuth et al., 1973). In the development of a strain energy function for the RBC membrane, Skalak et al. utilized a constant elastic modulus of 1 MPa, which was found to fit experimentally determined strain energy dissipation and membrane tension (Skalak et al., 1973). More recent techniques, namely dual beam optical stretcher and atomic force microscopy (AFM) have attempted to quantify the erythrocyte membrane elastic modulus (Bremmell et al., 2006; Bareil et al., 2007). While optical methods have largely agreed with older measurements, the elastic modulus determined from AFM was found to be 0.35–0.45 MPa and underestimates older techniques by approximately one order of magnitude (Bremmell et al., 2006). Our method approximated a Young's Modulus and Shear Modulus of approximately 1 MPa and 3 MPa, respectively, which agrees with those estimated from micropipette aspiration, rheologic methods, and optical stretching. We hypothesize that the discrepancy in elastic modulus relative to AFM is a consequence of the increase in elastic modulus in response to increase shear stress, as observed in capillary plug flow *in vivo*. Other mechanical properties traditionally used to asses erythrocyte membrane stiffness include the modulus of area compression, which is reported as the bulk modulus in our experiments. It is well-established that the modulus of area compression and elastic area compressibility are approximately one order of magnitude larger than both Young's Modulus and Shear Modulus (Evans et al., 1976). Our results demonstrate similar findings. In summary, erythrocyte membrane properties determined from our methods closely align with those from other methods despite the incorporation of experimentally measured dynamics observed during capillary plug flow in our method.

Determination of erythrocyte membrane wall shear stress is difficult due to spatial variability in membrane thickness. Membrane tension, defined as stress integrated over the membrane thickness, is therefore a more widely accepted metric. While membrane tension is generally assessed via micropipette aspiration, few studies have been able to obtain the spatial precision required to assess membrane tension during flow. Recently, Omori et al. reported a membrane tensions in the range of 10^−6^ and 10^−4^ N/m, with the upper threshold correlating with the minimum von Mises stress required for hemolysis (Omori et al., 2012). The membrane tension reported in this study support these findings. We attribute the increased range in our findings to errors in temporal averaging and discontinuities inappropriately corrected for in our numerical methods. As our findings for membrane tension agree with those presented in the literature, we hypothesize that the estimated wall shear stress is representative of shear stress observed *in vivo*. Of note in our study is the lack of statistically significant differences in wall shear stress, membrane tension, and shear strain. We hypothesize that during capillary plug flow, erythrocyte dynamics are constrained by the mechanical environment within the capillary due to the inability for active capillary dilation or constriction, a consequence of the lack of smooth muscle. Therefore, for RBCs in plug flow, the strain tensor must be conserved across all cells. The increase in mechanical properties by approximately one order of magnitude observed in the secondary heterogeneous population of RBCs identified by PCA is insufficient to result in significant changes in shear stress, thus accounting for the conserved stress among the populations of cells investigated.

The reticuloendothelial system (RES) is responsible for clearance of RBCs with membrane changes, known as eryptosis. Briefly, phagocytic macrophages in the RES mediate eryptosis and red cell clearance via complement and IgG dependent mechanisms (Halpern et al., 1957; Kelton, 1987). Consequently, the RES is responsible for clearance of schistocytes and hemolytic debris. However, recent studies have suggested that oscillatory flow conditions during splenic flow may induce vesiculation to increase RBC lifespan as a protective mechanism (Asaro et al., 2018). While it is well-established that this mechanism is responsible for the pathogenesis of membranopathies, namely hereditary spherocytosis, the protective mechanism of vesiculation for erythrocyte aging explains the increased flux through the RES during transfusion (Perrotta et al., 2008). Heterogeneous populations are identified by splenic macrophages and targeted for clearance. Consequently, the increased lifespan of RBCs exogenously introduced is likely a result. Vesiculation of transfused RBCs may be responsible for their increased lifespan *in vivo*. The results from this study demonstrate modest increase in membrane stiffness without changes in membrane wall shear stress and shear strain in erythrocyte mechanics analyzed in the experimental group. Our results suggest that protective vesiculation of transfused cells in the RES is responsible for altering erythrocyte membrane mechanics and dampening adverse mechanical injury from heterogeneous RBCs.

Strain was calculated by numerically determining discretized deformation gradient tensors for each pixel identified on the RBC membrane via image processing. To account for treadmilling, the weighting function employed to determine discretized deformation gradients spanned a substantial area of the erythrocyte membrane. Consequently, one of the main advantages of the methods presented here involved approximation of mechanical properties from experimentally estimated strains *in vivo*. Comparable methods to calculate shear strain and wall shear stress involve *in vitro* isolation of the erythrocyte or extensive modeling of erythrocyte mechanics. However, the methods presented here account for the unique environment to which the cell is subjected *in vivo*. As erythrocyte mechanics are shear dependent, we hypothesize that the mechanical properties estimated in these experiments are more representative of RBC mechanics in the circulation. The methodology developed in this study additionally has much utility of understanding erythrocyte mechanics in the context of various hematologic pathologies, namely sickle cell disease (SCD), membranopathies, and hemoglobinopathies, utilizing animal models *in vivo*. Clinically, the only treatment for membranopathies, like hereditary spherocytosis and elliptocytosis, is splenectomy (Perrotta et al., 2008). Understanding the mechanics of the RES and changes in mechanics at the level of the microcirculation may allow for the development of new therapeutics. Furthermore, in SCD, clinical management for vaso-occlusive crisis due to RBC agglutination and hypoxia involves opioid therapy, resulting in increased rates of addiction among these patients (Shapiro et al., 1997). Application of the methods presented in this study have the potential to discover new methods and therapies for acute vaso-occlusive crisis in SCD by understanding changes in erythrocyte mechanics and perfusion.

Limitations of this study include assumptions to account for viscoelasticity and shape memory, due to the short time scales observed, and of Hookean isotropy for the determination of the stress tensor. The strain energy function for erythrocyte membranes is non-linear as assessed by Skalak et al. (1973). In this model, we utilized Lagrangian coordinates to estimate the required mechanical properties given a pressure gradient. Rather than assuming that mechanical shape is coupled with flow, we assumed that the external pressure gradients required for flow mechanically constrain the shape of the erythrocyte membrane. Consequently, given an observed shape, we can estimate the mechanical properties. As a result of these assumptions as well as the 2D analytic methodology utilized, the quantified mechanical constants should be interpreted cautiously. Future directions should utilize the proper adjustments of the stress-strain relationships employed in this study and account for the viscoelastic properties of the membrane. Furthermore, the study assessed RBC mechanics by determining two-dimensional strain tensors at the imaging plane. Future directions should employ more sophisticated modeling and image processing techniques to extrapolate the three-dimensional deformation of the erythrocyte in the capillary. Due to the non-equivalence of 2D and 3D mechanical properties of erythrocytes, this is a significant limitation of this study. While our results demonstrate the presence of a secondary heterogeneous population of cells with different mechanical properties identified by PCA, we are unable to conclude that the rigid cells were indeed the transfused cells. Future directions should aim to identify the etiology and biological mechanisms responsible for the heterogeneity quantified.

## 5. Conclusion

The study presented here quantified the mechanical properties and wall shear stress experienced by RBC membranes during capillary plug flow *in vivo* utilizing intravital imaging and computational methods. Erythrocyte mechanical properties were determined via numerical methods utilizing the discretized displacement gradient tensor across the erythrocyte membrane over time and the Navier-Lame equations of elastodynamics. To our knowledge, the methods presented here are the first erythrocyte mechanical properties and shear stress *in vivo* during capillary plug flow. The methodology developed in this study additionally has much utility of understanding erythrocyte mechanics in the context of various hematologic pathologies, namely sickle cell disease (SCD), membranopathies, and hemoglobinopathies. Future directions should aim to confirm the findings presented here and apply them to investigate pathologies with impaired erythrocyte mechanics.

## Data Availability Statement

The raw data supporting the conclusions of this article will be made available by the authors, without undue reservation, to any qualified researcher.

## Author Contributions

All authors listed have made a substantial, direct and intellectual contribution to the work, and approved it for publication.

### Conflict of Interest

The authors declare that the research was conducted in the absence of any commercial or financial relationships that could be construed as a potential conflict of interest.
